# Pregnant Women’s Experiences of the Conditions Affecting Marital Well-Being in High-Risk Pregnancy: A Qualitative Study

**DOI:** 10.30476/ijcbnm.2020.85666.1285

**Published:** 2020-10

**Authors:** Kobra Mirzakhani, Talaat Khadivzadeh, Farhad Faridhosseini, Abbas Ebadi

**Affiliations:** 1 Nursing and Midwifery Care Research Center, Mashhad University of Medical Sciences, Mashhad, Iran; 2 Department of Midwifery, School of Nursing and Midwifery, Mashhad University of Medical Sciences, Mashhad, Iran; 3 Psychiatry and Behavioral Sciences Research Center, Mashhad University of Medical Sciences, Mashhad, Iran; 4 Behavioral Sciences Research Center, Life style institute, Baqiyatallah University of Medical Sciences, Tehran, Iran; 5 Department of Nursing Management, Baqiyatallah University of Medical Sciences, Tehran, Iran

**Keywords:** High-risk, Marital, Pregnancy, Relationship, Well-being

## Abstract

**Background:**

High-risk pregnancy is associated with many problems which can affect marital well-being as well as maternal and fetal health. Yet, there is limited information about the conditions which affect marital well-being in high-risk pregnancy. This study aimed to explore the pregnant women’s experiences of the conditions affecting marital well-being in high-risk pregnancy.

**Methods:**

This qualitative study was conducted from October 2018 to December 2019. Participants were 24 women with high-risk pregnancy who were purposively selected from three public and two private hospitals as well as a primary healthcare center in Mashhad, Iran. Face-to-face semi-structured interviews were conducted for data collection. Data were analyzed concurrently with data collection through Graneheim and Lundman’s content analysis (2004). The MAXQDA program (v. 10) was used for data management.

**Results:**

Conditions affecting marital well-being in high-risk pregnancy were categorized into eleven subcategories and three main categories, namely emotional spousal intimacy in the midst of danger, husband’s commitment to manage the difficult conditions of pregnancy and sexual relationship during high-risk pregnancy.

**Conclusion:**

Several conditions can affect marital well-being in high-risk pregnancy. Healthcare providers can develop and use strategies for the effective management of these conditions, thereby improving marital well-being among women with high-risk pregnancy.

## INTRODUCTION

Pregnancy is a physiological process with different physical, mental, and social change. It may turn from a physiological process or low-risk condition to a high-risk condition if the pregnant woman or her fetus is at risk for loss. High-risk pregnancy (HRP) may be due to problems rooted in the pre-pregnancy or the pregnancy periods. ^1^ The prevalence of HRP in Iran and other countries is 25.6-75.6%. ^2 , 3^

HRP is associated with many different problems. For instance, it can cause physical and emotional tensions, anxiety, and depression. The prevalence rates of depression and anxiety in HRP are 12.5-44.2% and 16.9-54%, respectively. ^4^ Moreover, women with HRP are exposed to sociocultural problems and financial strains; hence, they experience problems in personal and family lives and insufficiency in role performance. ^5^ Psycho-emotional problems in HRP can negatively affect marital well-being (MWB) and marital relationship. ^6^

MWB is a multidimensional concept consisting of marital satisfaction, marital stability, marital commitment, and marital closeness. ^7^ Good MWB is associated with positive pregnancy-related experiences such as love, care, and satisfaction. MWB directly and indirectly affects mental happiness which is a mediating factor between marital relationship and mental health. ^8^ Therefore, it can improve pregnant women’s mental health, protect them against anxiety and depression, ^9 , 10^ and reduce the risk of suicide by 25%. ^10^ Better martial relationship and greater marital satisfaction are associated with a healthier lifestyle, higher quality of life, greater well-being, ^11^ and better physical and mental health. ^12^ Greater MWB and higher satisfaction with marital relationship reduce the serum levels of inflammatory factors, thereby improving physical health. ^13^ Beside maternal health, MWB in pregnancy can affect the fetal and child health. A study reported stressful events and marital dissatisfaction during pregnancy as significant predictors of infectious diseases among children during the first year of life. These diseases have long-term effects on the immune system development and increase the risk of developing allergy and asthma later in life. Contrarily, effective management of marital relationship reduces the risk of infectious diseases among children after birth. ^14^

Many different conditions can affect MWB and its contributing conditions among pregnant and non-pregnant women. ^15^ These factors or conditions among non-pregnant women include demographic characteristics, personality traits, attachment style, intimacy, couples’ families, family counselors and therapists, sexual relationship, ^16^ fear associated with sexual relationship, ^17^ pathological conditions, and educational interventions. ^16^ According to research, these conditions are context-bound and may differ from one context to another. ^12^ Nonetheless, no study had yet evaluated these conditions in Iran. The qualitative approach allows the researcher to interpret and better understand the experiences of women with HRP respecting conditions affecting MWB in HRP and Iran’s socio-cultural context. Therefore, the present study was conducted to explore pregnant women’s experiences of the conditions affecting marital well-being in high-risk pregnancy. 

## METHODS

This qualitative study was conducted from October 2018 to December 2019 using conventional content analysis. Study setting was HRP care wards and clinics of three public hospitals (including Imam Reza, Qaem, and Ommolbanin) and two private hospitals (namely Mehr and Pasteur) as well as a primary healthcare center, all in Mashhad, Iran. The study population comprised all pregnant women diagnosed with HRP based on the guideline of the National Institute for Health and Care Excellence, ^1^ who had been registered in Medical Care Monitoring Center system. This system aimed to monitor women with HRP and their problems and facilitate access to them. Eligible women for the study were purposively selected with maximum variation regarding their age, gestational age, socioeconomic status, and type of pregnancy complication (Table 1). Eligibility criteria were agreement for participation in the study, ability to communicate in Persian, and ability to share HRP-related experiences. Exclusion criterion was widowed women.

**Table 1:** Characteristics of the participants included in the study

Participant	Age (Year)	Educational level	Occupation	Gravidity	Number of live children	Gestational age(Week)	Type of pregnancy complication
1	30	Primary education	Housewife	4	0	36	Preeclampsia
2	34	Bachelor’s degree	Painter	2	1	28	Placenta previa and accreta
3	31	Master’s degree	PhD Candidate	1	0	7	Hyperemesis gravidarum
4	41	Primary education	Housewife	1	0	27	Angioedema and suspected lupus
5	36	Master’s degree	PhD Student	2	1	32	Gestational diabetes mellitus; twin pregnancy
6	40	Bachelor’s degree	Housewife	3	0	31	Premature rupture of membranes, preterm labor, ^ivf^
7	35	Diploma	Driving instructor	3	2	36	Cardiac problem and diabetes mellitus
8	28	Diploma	Housewife	2	0	26	Hypothyroidism and premature rupture of membranes
9	30	Secondary education	Housewife	2	1	25	Premature rupture of membranes
10	37	Bachelor’s degree	Accountant	6	0	29	Premature rupture of membranes, Cerclage ^ivf^
11	30	Diploma	Housewife	2	1	36	Oligohydramnios
12	32	Master’s degree	Teacher	2	0	10	Pneumonia and respiratory distress
13	36	Bachelor’s degree	Dress designer	2	1	36	Fever and pneumonia
14	30	Master’s degree	Teacher	2	0	37	Severe depression and anxiety
15	46	Primary education	Housewife	4	3	29	Mitral stenosis
16	30	Master’s degree	Lawyer	1	0	33	Leukemia
17	22	Diploma	Housewife	2	1	10	Pyelonephritis
18	33	Bachelor’s degree	Teacher	1	0	25	Cholestasis
19	44	Primary education	Carpet weaver	1	0	30	Hypertension and Epigastric Pain
20	38	Diploma	Carpet weaver	1	0	12	Multiple pregnancy
21	32	Secondary education	Housewife	2	1	30	Preterm labor and twin pregnancy
22	36	Primary education	Housewife	6	5	13	^dvt^
23	23	Diploma	Housewife	2	0	30	Aortic stenosis
24	26	Secondary education	Housewife	2	1	38	Thrombocytopenia

ivf: in vitro fertilization; dvt: Deep Venous Thrombosis

Data were collected through semi-structured interviews held by the first author. The place of each interview was determined according to the interviewee’s preference and was a quiet and comfortable room either in the study setting, her house, or any other desired place. Interview questions were on the participants’ experiences and feelings about MWB and its contributing conditions. Sampling and data collection were continued up to data saturation, which was achieved after interviewing 21 participants. Yet, three more interviews were conducted to ensure saturation, which produced no new data. The first, third, and fifth participants were interviewed twice. Finally, data collection was finished with 27 interviews with 24 participants. Based on the participants’ information and conditions, the length of the interviews varied from 30 to 75 minutes (with a mean of 45 minutes). All interviews were completely recorded with the participants’ consent using an MP3 recorder. Data were analyzed using the qualitative content analysis suggested by Graneheim and Lundman (2004). ^18^

Interviews were considered as units of analysis. After each interview, the first author listened to it several times to obtain a general understanding about it, and then typed it word by word. Sentences or paragraphs related to the study aim were identified as the meaning units. Then, meaning units were reviewed for several times and coded. During the processes of data reduction and condensation, similar codes were grouped together to generate subcategories. Subcategories were also grouped to generate larger categories. The MAXQDA program (v. 10, VERBI Software GmbH, Berlin) was used for data management.

Trustworthiness of the data was ensured through four criteria proposed by Lincoln and Guba (1985), namely credibility, dependability, confirmability, and transferability. ^19^ To ensure credibility, the first author, who performed data collection and analysis, was continuously engaged with the data even during her daily activities. Moreover, during member checking, the coded texts together with their corresponding codes were reviewed by coauthors who confirmed the accuracy of the data analysis. Three participants also confirmed the accuracy of data with the generated codes. They confirmed the congruence between the codes and their own experiences. To ensure dependability, the voice files and the transcripts of the interviews were provided to three qualitative researchers and they were asked to independently code the interviews. Their findings were similar to our results. Confirmability was ensured through documenting all phases of the data analysis in order to provide others with the opportunity to assess and confirm the accuracy of the data analysis. Transferability was also maintained through sampling with maximum variation and providing detailed description of the process, and data about the participants’ age, educational level, number of pregnancies, type of pregnancy complication, and gestational age.

The Ethics Committee of Mashhad University of Medical Sciences, Mashhad, Iran, approved the study (code: IR.MUMS.NURSE.REC.1397.039). All the ethical issues of qualitative studies were considered. Before each interview, the aim of the study and confidentiality of the information was explained to the interviewee and her written informed consent was obtained. In addition, the participants had the right to withdraw from the study at any time. 

## RESULTS

In total, 24 women with HRP participated in the study. Their mean age was 32.87±12.2 years with a range of 22–46. Their gestational age ranged from seven to 38 weeks. The participants 8 (33.33%) had primary or secondary education, 6 (25%) had diploma, 5 (20.83%) had Bachelor’s degree, and 5 (20.83%) had either master’s degree or were PhD student. The participants 11 (45.83%) were housewife and 13 (54.17%) were employed. The number of participants’ children ranged from zero to five. The participants 20 (50%) had no children and 20 (50%) had one or more children alive (Table 1).

During data analysis, conditions affecting MWB in HRP were grouped into eleven subcategories and three main categories, namely emotional spousal intimacy in the midst of danger, husband’s commitment to manage the difficult conditions of pregnancy, and sexual relationship during HRP (Table 2). 

**Table 2:** Subcategories and categories emerged related to conditions affecting marital well-being in high risk pregnancy

Subcategories	Categories
Intimate relationship in the shadow of couples’ personalities	Emotional spousal intimacy in the midst of danger
Emotional closeness	
Physical closeness	
Confidence in the continuity of marital relationship	
Husband’s physical support	Husband’s commitment to manage the difficult conditions of pregnancy
Husband’s emotional support	
Husband’s financial support	
Assigning high priority to pregnant women’s health	
Stress related to the discontinuation of sexual relationship	Sexual relationship during HRP
Forgotten sexual relationship	
Purposefulness of sexual relationship	

### 1. Emotional Spousal Intimacy in the Midst of Danger

This main category described the participants’ emotional intimacy with their spouses despite their HRP-related problems and showed that MWB in HRP depended on an intimate spousal relationship. Such relationship gave participants good feelings, satisfaction, calmness, and hope and reduced their discomfort, stress, and anxiety. The four subcategories of this main category were intimate relationship in the shadow of couples’ personalities, emotional closeness, physical closeness, and confidence in the continuity of marital relationship.

### 1.a. Intimate Relationship in the Shadow of Couples’ Personalities

Most participants considered the personality traits of themselves and their spouses as significant conditions affecting intimacy and good feelings in the difficult conditions of HRP. Husband’s positive personality traits such as patience, self-sacrifice, kindness, honesty, and calmness helped them have good feelings and experience MWB. On the contrary, the husband’s negative personality traits such as being sharp-tongued and indifferent negatively affected the participants’ MWB and their intimate relationship with their husbands.

*"My husband is really calm. His kindness and self-sacrifice help me feel good despite my pregnancy-related problems (32-year-old; Second pregnancy with pneumonia and respiratory distress).*"

Some participants also noted that their own personality traits such as patience, adaptability, and resilience helped them cope with their HRP and its associated problems. These participants not only did not consider their husbands as the cause of the problems they experienced during pregnancy, but also had compassion towards them and considered them as the victims of the difficult conditions of HRP. The distresses of HRP less frequently affected the MWB of these participants and they were able to protect their intimate relationship with their husbands in the critical conditions of HRP. 

*"I’m a patient and adaptable person. I attempt not to have irrational expectations from my husband in order not to harm our relationship and not to add to my stress (34-year-old; second pregnancy with placenta previa and accreta)."*

### 1.b. Emotional Closeness

Emotional closeness was another factor affecting MWB in HRP. Most participants received calmness mostly from their husbands and noted that they could not substitute anybody else for their husbands. Emotional closeness rooted in husband’s kind and honest relationship, comradeship, and empathy. Participants considered their husbands’ comradeship and empathy with them as conditions which reduced their mental strains, strengthened their relationship with their husbands, and gave them greater satisfaction with their physical conditions.

"My husband’s good conduct helps me feel good. When he enters home, he greets me with smile despite fatigue in his face. I also feel good when he talks with me (40-year-old; third pregnancy with premature rupture of membranes)." 

Some participants noted that HRP promoted their emotional closeness and resulted in their husbands’ greater attention to their needs and greater kindness towards them. Moreover, the necessity to abandon their social and occupational activities and stay more at home to have bed rest had provided them with ample opportunity to talk with their husbands; therefore, they had strengthened their attachment to their husbands. Yet, intimate spousal relationship could also negatively affect MWB in HRP because HRP-associated problems made the husbands sad and concerned with their wives’ health. Such sadness and concern negatively affected the pregnant women and caused them to feel guilty. On the other hand, the husbands’ sarcastic behaviors and women’s senses of being forgotten and rejected by their husbands negatively affected perceived MWB in HRP.

### 1.c. Physical Closeness

Most participants noted that their husbands’ physical presence calmed them and reduced their stress, depression, and discomfort. Therefore, they liked their husbands to be with them when they were hospitalized. The participants who were deprived of their husbands’ physical presence due to hospitalization or staying at their fathers’ houses experienced stress and dissatisfaction because they missed their husbands and were concerned about their husbands’ loneliness.

*"Now, I’m hospitalized and miss my husband, my life, and my house. My husband is alone at home. I’m preoccupied with him and how he manages loneliness at home (36-year-old; second pregnancy with gestational diabetes mellitus; twin pregnancy).*"

### 1.d. Confidence in the Continuity of Marital Relationship

Confidence in the continuity of marital relationship, despite having an HRP, was a significant factor affecting the participants’ spousal intimacy and MWB. Some participants felt weak and insufficient in performing their roles; hence, they felt that their marital relationship was moving toward separation, particularly if these feelings of weakness and insufficiency were evoked by their husbands or their husbands’ families. Some participants were even concerned about their husbands’ second marriage due to their perceived insufficiency.

*"My mother-in-law says that I cannot have a normal pregnancy, always feel ailment during pregnancy, and annoy her son; hence, she warns me against another pregnancy. She says that for having another child, my son is more comfortable to have another wife. Such sayings can affect my husband (30-year-old; fourth pregnancy with preeclampsia and without a live child).*"

### 2. Husband’s Commitment to Manage the Difficult Conditions of Pregnancy 

The second category of the study was husband’s commitment to manage the difficult conditions of pregnancy. This category showed that only those women could perceive high level of MWB whose husbands felt responsible towards their wives’ problems, were committed to promote their health, took measures to prevent the aggravation of HRP-related problems, assigned high priority to the maintenance of their wives’ health, and attempted to help them cope with HRP. This main category had four subcategories, namely husband’s physical support, husband’s emotional support, husband’s financial support, and assigning high priority to pregnant women’s health.

### 2.a. Husband’s Physical Support

The participants reported that they felt well and healthy if their husbands accompanied them in antenatal classes, helped them perform household, family care and childrearing activities, engaged in HRP-related decision making, attempted to receive the best type of care services and gain others’ support, and prevented others from interfering in pregnancy-related affairs.

"In general, my husband had a good conduct in my pregnancy and actively supported me. For example, if I wanted to go to a laboratory , he got a leave and accompanied me and said that I’m more important than any other thing. These behaviors gave me good feelings. He wholeheartedly supported me (30-year-old; second pregnancy with oligohydramnios)." 

### 2.b. Husband’s Emotional Support

Husband’s emotional support was another factor affecting MWB among women with HRP. Husbands who ensured their wives about their ongoing support gave them feelings of security, assurance, and hope. Emotional support was of greater importance in the case of pregnant women’s feelings of frustration and helplessness.

*"I felt disappointed when they said that my blood pressure was high and my and my baby’s life was at risk. But, my husband supported me, gave me hope, said that God supports me, and said that he would do his best in order to prevent any damage to me and my baby (41-year-old; first pregnancy with angioedema and suspected lupus).*"

### 2.c. Husband’s Financial Support

The participants reported that they felt assured and secured if their husbands ensured them that they would cover all costs related to diagnostic and therapeutic procedures in HRP. Insurance coverage and other types of financial support also prevented the participants from experiencing stress. Contrarily, poverty, inability to afford HRP-related costs, and living in a rented house, and husband’s limited attempt to reduce the family’s financial problems led to their stress and negatively affected their MWB.

*"We have not yet experienced any problem related to the costs of pregnancy because my husband has supplemental health insurance which pays all of pregnancy-related costs (35-year-old; third pregnancy with cardiac problem and diabetes mellitus).*"

### 2.d. Assigning High Priority to Pregnant Women’s Health

The participants whose husbands assigned high priority to their health and prioritized fulfillment of their needs over other activities reported higher levels of perceived MWB and satisfaction. On the contrary, they experienced suffering if they felt that their husbands paid attention to them mostly to ensure their babies’ health.

*"My husband always says that the most important thing is my health. He says that his recommendations are firstly for the sake of my health and then my child. These behaviors give me good feelings (31-year-old; first pregnancy with hyperemesis gravidarum).*"

### 3. Sexual Relationship During HRP

The third category of the study was sexual relationship during HRP. The participants reported that the limitation or discontinuation of sexual relationship had influenced their MWB. The three subcategories of this category were stress related to the discontinuation of sexual relationship, forgotten sexual relationship, and purposefulness of sexual relationship.

### 3.a. Stress Related to the Discontinuation of Sexual Relationship

The participants and their husbands had limited sexual relationship or avoided it due to their fear over inflicting damage on themselves, their fetuses, and their pregnancy. Discontinuation or limitation of sexual relationship was associated with distress and feeling of guilt for participants because they felt that it had resulted in emotional separation from their husbands. On the other hand, they felt guilty due to their inability to fulfill their husbands’ sexual needs. Moreover, because of their inability to engage in sexual relationship or their fear over its associated probable damages, some of them felt that they had lost their femininity or were under their husbands’ pressure and sarcasm. Some participants were also concerned with their husbands’ probable extramarital relationships. All these fears, concerns, and distresses negatively affected their MWB.

*"We didn’t have sexual relationship during pregnancy at all. I said to myself that he is a man and has his own sexual needs. I thought that the suppression of his sexual needs over the one-year period of pregnancy and postpartum can place him under considerable pressure though it may not create considerable pressure for a woman. This problem annoyed me (40-year-old; Third pregnancy with premature rupture of membranes).*"

### 3.b. Forgotten Sexual Relationship

Most participants noted that HRP was associated with their husbands’ intense involvement in HRP-related problems and household activities, thereby causing them fatigue and resulting in mutual reluctance and inability to have sexual relationship. Moreover, physicians and midwives had recommended them to avoid sexual relationship without providing them with any sexuality counseling.

"My husband was so intensely involved in my pregnancy that he had no time for thinking about his sexual needs. My poor husband wakes up early in the morning and hastily performs the chores, takes our child to the nursery, and then, goes to his work. After that, he should pick up the child from the nursery and serve his food and so on, until he sleeps at night with extreme fatigue (34-year-old; second pregnancy with placenta previa and accrete )."

### 3.c. Purposefulness of Sexual Relationship

In most cases, HRP had changed the participants’ and their husbands’ goals and priorities, so that their main goal was to protect pregnancy and fetal health. Such changes had resulted in postponing other goals and activities, such as sexual relationships, to the postnatal period. They considered protecting pregnancy, fetal health, and maternal health more important than sexual relationship and had accepted its discontinuation until childbirth.

*"I got pregnant after eight years of infertility and three unsuccessful in-vitro fertilizations. Therefore, we avoided sexual relationship. My doctor also had warned me against it. It was annoying and I felt bad over it. But, we accepted it for the sake of the baby. Sometimes, I suggested sexual relationship to my husband, but he rejected it in order to protect the pregnancy (40-year-old; Third pregnancy with premature rupture of membranes).*"

## DISCUSSION

This qualitative study aimed to explore the experiences of women with HRP with respect to conditions affecting MWB in HRP. Our findings are in line with previous studies; a study reported that the six main aspects of marital relationship were romance, respect, trust, finance, meaning, and physical intimacy. ^20^ Another study showed that MWB had the four main dimensions of marital satisfaction, marital stability, marital commitment, and marital closeness. ^7^

Study findings indicated that MWB was affected by intimate and friendly emotional relationship in the midst of danger. Intimate relationship is a key factor affecting MWB. It can reduce depressive symptoms ^21^ which are a component of poor MWB. ^7^ Intimate relationship also provides opportunities for experiencing intimacy and love and sharing positive experiences, helps abandon discomforting psychological experiences such as anxiety, anger, and grief, enhances the ability to tolerate distress, and results in marital satisfaction and MWB. ^22^ As psychological distress is a component of poor MWB and HRP is a stressful experience associated with threat and probable loss, ^23^ intimate and friendly relationship in HRP can enhance MWB.

The couple’s personalities and physical and emotional closeness were also found as key conditions affecting marital relationship and MWB. The husbands’ positive personality traits are a protective factor against marital stress among women and positively affect the quality of marital and familial relationships. Personality traits such as humility, humanity, kindness, and forgiveness have significant positive correlations with marital satisfaction and MWB. Compassion, defined as understanding others’ suffering and having motivation for its alleviation, also can bring others happiness and improve their well-being. ^24^ Compassion, warmth, and kindness facilitate supportive responses in interpersonal relationships among the couples. ^25^ On the contrary, those with limited compassion have limited understanding of others’ suffering, make no serious effort for its alleviation, are considered as indifferent and irresponsible, and can cause marital dissatisfaction and poor MWB. Therefore, lack of compassion is among the most common causes of requesting family counseling services. ^26^ A study also reported that the husbands’ negative personality traits and unfriendly and non-empathetic relationships are associated with disappointment and poor well-being among pregnant women with severe perinatal depression. ^27^

We also found confidence in the continuity of marital relationship as a factor contributing to MWB in HRP. One of the fears and sources of stress among women with HRP is their fear over rejection by their husbands and subsequent marital separation. ^28^ Fear and stress can threaten well-being, while confidence and assurance can relieve stress. ^29^ Moreover, confidence in the continuity of marital relationship contributes to happiness, satisfaction, and well-being. ^7^ Loyalty, respect, and confidence are important aspects of satisfactory and successful marital relationships. ^20^

The study findings also showed that another factor affecting MWB among women with HRP was husband’s commitment to manage the difficult conditions of HRP and protect pregnancy through providing physical, emotional, and financial support. Perceived respect and commitment in marital relationship can enhance marital satisfaction and strengthen marital relationship. ^30^ The couples’ commitment to enhance marital satisfaction and MWB is the most important factor affecting MWB. The husband’s commitment to pregnancy is manifested in his support for his wife. Support in turn creates a sense of security, reduces stress, thereby improving well-being. ^31^ Other studies also reported that the husband’s support reduces anxiety, stress, pregnancy-related concerns and the risk of post-traumatic stress disorder, and improves perceived MWB and mental health among women with HRP. ^32 , 33^ Social support and intimate marital relationship also reduce the serum level of inflammatory factors and enhance cardiovascular health. ^13^ On the contrary, lack of husband’s support increases the risk of preterm delivery and small-for-gestational-age baby. ^34^ In Eric Fromm’s theory, the components of love are respect, responsibility, attention, recognition, and support. Accordingly, a committed relationship in which couples feel responsible towards each other and support each other in case of problems is associated with greater love, satisfaction, happiness, and confidence. ^35^

Findings also indicated the husband’s financial support for pregnancy as another aspect of his commitment to protect pregnancy and a factor affecting MWB in HRP. A study showed that the husband’s financial support for pregnancy is associated with greater resilience and higher perceived well-being among pregnant women who are at risk for preterm delivery. ^36^ However, poor financial status can directly and indirectly contribute to the development of depressive symptoms and poor well-being in pregnancy. ^37^

We also found sexual relationship as a main factor affecting MWB in HRP. Most participants had temporarily limited or discontinued their sexual relationships due to the profound effects of HRP and its associated problems on their physical and mental health or due to their fear over inflicting damage on pregnancy. Negative attitude towards sexual relationship during pregnancy is not unique to women; rather, a study reported that more than half of the men have negative attitudes towards sexual relationship during pregnancy. ^38^ Although sexual relationship is limited during pregnancy, all pregnant women feel deeper attachment to their spouses and need greater emotional attention. ^39^ A study reported fear as the most important reason for avoiding sexual relationship among the pregnant women. That study also reported that women who avoided sexual relationship due to fear were more likely to show the symptoms of fetal distress; hence, interventions which reduce fear over sexual relationship during pregnancy are needed to reduce the couples’ concerns and anxiety. ^17^

Most participants noted that their physicians or midwives had recommended them to limit or discontinue sexual relationship. Yet, they had not received any sexuality counseling from their physicians or midwives probably due to the fact that the Iranian culture disapproves straight talks about sexual issues. ^40 , 41^ Avoidance from talking about sexual issues and concerns may prevent the early diagnosis and the effective management of sexual problems. Given the great need of pregnant women for intimacy ^10^ and its positive effects on MWB, educational interventions are needed to better manage sexual relationship during pregnancy. ^8^ Moreover, medical recommendations for limiting and discontinuing sexual relationships during pregnancy should be provided based on firm scientific evidence. 

The strengths of this study were its qualitative design and data collection through semi-structured interviews. Qualitative approach can help view the data more extensively and deeply about of the conditions affecting MWB in HRP that was reported for the first time. Among the limitations of the study was data collection only from pregnant women not their husband’s participants. Future studies are recommended to explore the concept of MWB in HRP based on the experiences of both pregnant women and their husbands. Further studies are also needed to explore and compare MWB in HRP, low-risk pregnancy, and postpartum period.

## CONCLUSION

This study suggests that the most important conditions affecting MWB in HRP are emotional spousal intimacy in the midst of danger, husband’s commitment to manage the difficult conditions of pregnancy, and sexual relationship during HRP. HRP is a stressful experience associated with threat and probable loss. Therefore, effective measures and strategies are needed to manage the conditions which can affect MWB, thereby improving MWB during HRP. Strategies such as doing educational interventions and counseling for couples in high-risk pregnancies, paying attention to the conditions of the couple’s closeness in the case of hospitalization, having bed rest in high-risk pregnancies and providing evidence-based care are recommended. 
